# Salinity Changes the Dynamics of Pyrethroid Toxicity in Terms of Behavioral Effects on Newly Hatched Delta Smelt Larvae

**DOI:** 10.3390/toxics9020040

**Published:** 2021-02-20

**Authors:** Amelie Segarra, Florian Mauduit, Nermeen R. Amer, Felix Biefel, Michelle L. Hladik, Richard E. Connon, Susanne M. Brander

**Affiliations:** 1 Anatomy, Physiology & Cell Biology, School of Veterinary Medicine, University of California, Davis, CA 95616, USA; fmauduit@ucdavis.edu (F.M.); rnermeen@sci.cu.edu.eg (N.R.A.); fkbiefel@ucdavis.edu (F.B.); reconnon@ucdavis.edu (R.E.C.); 2 Department of Entomology, Faculty of Science, Cairo University, Giza 11311, Egypt; 3 Aquatic Systems Biology Unit, Department of Ecology and Ecosystem Management, Technical University of Munich, Mühlenweg 22, D-85350 Freising, Germany; 4 US Geological Survey, California Water Science Center Sacramento, Sacramento, CA 95819, USA; mhladik@usgs.gov; 5 Department Fisheries and Wildlife, Coastal Oregon Marine Experiment Station, Oregon State University, Corvallis, OR 97331, USA; susanne.brander@oregonstate.edu

**Keywords:** bifenthrin, permethrin, estuary, multiple stressors, behavior, fish, pesticide, thigmotaxis, endangered species, global climate change

## Abstract

Salinity can interact with organic compounds and modulate their toxicity. Studies have shown that the fraction of pyrethroid insecticides in the aqueous phase increases with increasing salinity, potentially increasing the risk of exposure for aquatic organisms at higher salinities. In the San Francisco Bay Delta (SFBD) estuary, pyrethroid concentrations increase during the rainy season, coinciding with the spawning season of Delta Smelt (*Hypomesus transpacificus*), an endangered, endemic fish. Furthermore, salinity intrusion in the SFBD is exacerbated by global climate change, which may change the dynamics of pyrethroid toxicity on aquatic animals. Therefore, examining the effect of salinity on the sublethal toxicity of pyrethroids is essential for risk assessments, especially during the early life stages of estuarine fishes. To address this, we investigated behavioral effects of permethrin and bifenthrin at three environmentally relevant concentrations across a salinity gradient (0.5, 2 and 6 PSU) on Delta Smelt yolk-sac larvae. Our results suggest that environmentally relevant concentrations of pyrethroids can perturb Delta Smelt larvae behavior even at the lowest concentrations (<1 ng/L) and that salinity can change the dynamic of pyrethroid toxicity in terms of behavioral effects, especially for bifenthrin, where salinity was positively correlated with anti-thigmotaxis at each concentration.

## 1. Introduction

The Delta Smelt, *Hypomesus transpacificus*, is a pelagic fish species endemic to brackish and freshwater portions of the Sacramento River and the San Francisco Estuary (California, USA). This fish has a migratory annual life cycle which follows the seasons and where life stages vary spatially across the San Francisco Bay Delta (SFBD). Delta Smelt have a complex life cycle [1], with adults generally migrating upstream to spawn in freshwater (0.5 Practical Salinity Unit; PSU), where resulting larvae remain until they become juveniles. During late spring, they begin their migration downstream to low-salinity zones (1–6 PSU), where they continue to mature until the next winter [2]. The Delta Smelt has experienced a dramatic decline in abundance since the 1980s [2,3]. It was first classified as “threatened” under the Federal and State Endangered Species Act (ESA) in 1993, then listed as “endangered” under the California Endangered Species Act (ESA) in 2009 [4], and was finally classified as “critically endangered” by the International Union for Conservation of Nature 5 years later [5]. The listed threats for the Delta Smelt’s decline are multiple and include natural habitat modification and degradation (e.g., dams and water diversions), introduced species competition and predation, decreased food availability, changes in the physiochemical properties of the system associated with global climate change [2,6], and pollution not only from domestic and industrial wastewater, but also from agricultural and surface runoff [7,8,9,10,11].

Pyrethroid insecticides are heavily used in agricultural and urban areas, and are commonly detected in waterbodies worldwide. In the San Francisco Bay Delta (SFBD) estuary, their concentrations increase during the rainy season (November to April) due to runoff, coinciding with the spawning season of Delta Smelt (December to May) [12]. Among these insecticides, the frequent detection of two pyrethroids, permethrin and bifenthrin, which are highly toxicity to aquatic life, is of great concern for the Delta Smelt habitat and overall fitness for species living in this system [12,13,14]. Permethrin and bifenthrin are frequently detected in the low ng/L to µg/L range in sediment and surface waters [15,16]. For example, bifenthrin has been measured at concentrations up to 106 ng/L upstream of known Delta Smelt habitats [12,17]. Given the lipophilic nature of pyrethroids, it is expected that, in fish, these insecticides are widely absorbed and bioaccumulated in tissues with a high lipid content, including fat, central and peripheral nervous [18], and larval yolk-sacs. The mechanism of action of both permethrin and bifenthrin is through binding to voltage-gated sodium ion channels (VGSCs), delaying closure of the channel and resulting ultimately in convulsions and death [19,20]. Pyrethroids are also reported to be more toxic to aquatic organisms than terrestrial organisms due to their effect on Na^+^ATPase, an enzyme involved in osmoregulation [21]. In fish, the pyrethroid cypermethrin can reduce sperm motility and fertilization success [22,23]. Permethrin and bifenthrin have both been shown to impact endocrine activity, acting as an estrogen receptor agonist [24]. Exposure to bifenthrin (0.5 ng/L) altered endocrine signaling and reduced the number of fertilized eggs per female in Inland Silverside, *Menidia beryllina*, a euryhaline fish species whose habitat overlaps with Delta Smelt [25]. Pyrethroid metabolites are reported as having a greater endocrine activity than the parent molecules in in vitro tests [26,27,28]. Estrogen and estrogen mimics exposure during the larvae period can influence the sex ratio of fishes [29], suggesting that early life exposure to these low concentrations of pyrethroids may indirectly precipitate fish population decline [30,31]. Given this knowledge, it is not surprising that pyrethroids have been postulated to have played a significant role in the decline of several fish species in the California Bay-Delta [10], but such cause–effect relationships are extremely difficult to demonstrate in field populations.

Salinity is an important factor in the Delta Smelt life cycle and influences their spatial distribution especially during the spawning period. However, climate change impacts are predicted to alter abiotic conditions, which may potentially increase environmental stress on estuarine species. Climate change-related increased duration/frequency of drought periods reduces the freshwater entering estuaries and, with coupled sea level rise, this allows for intrusions of higher-salinity water from the ocean, pushing the low-salinity zone further upstream [32,33]. This further compress Delta Smelt habitat and relative distribution. Salinity has also been shown to interact with chemical properties of organic pesticides by decreasing the water solubility of the compound and therefore potentially modulating their toxicity [34]. Pyrethroids are highly hydrophobic and studies have shown that the more hydrophobic a contaminant is, the larger the impact salinity has on its affinity for a solid phase such as sediments and/or tissues [35]. Moreover, the fraction of pyrethroid insecticides in the aqueous phase (with either no or limited suspended sediments present) increases with increasing salinity, potentially increasing the risk of exposure for aquatic organisms at higher salinities [36]. Therefore, examining the effect of salinity on the toxicity of two frequently detected pyrethroid insecticides, permethrin and bifenthrin, is essential in assessing the risk that these insecticides pose to estuarine fish, especially during sensitive stages of development (i.e., yolk-sac larvae).

Locomotion behavior is key for the survival of animals, and it is governed by biotic and abiotic factors. The observation of behavioral change at environmentally relevant concentrations of contaminants in fish larvae may provide a strong indicator for neuroactivity effects. Behavioral endpoints are highly sensitive and ecologically relevant to assess these effects [23]. The potential to identify interactions of chemicals with the nervous system using behavioral assays has been extensively used in model fish species such as Zebrafish (*Danio rerio*) [37], where larvae are often used for studying the behavioral toxicity of environmental contaminants [38,39,40]. Among the several locomotor behavioral assays, the light and dark locomotion test, in which various swimming activity endpoints are measured, has been used to evaluate and screen numerous compounds for potential neuroactive properties [41,42]. At sublethal concentrations, pyrethroids are found to induce changes in the protein metabolism and/or behavior in larval fish such as Zebrafish, Inland Silversides and Delta Smelt [43,44,45,46,47,48]. Mundy et al. (2020), have recently adapted a light and dark cycle behavioral test, routinely used on Zebrafish species, for Delta Smelt larvae and used it to assess sensitive alterations in swimming activity due to environmentally relevant pyrethroid concentrations [47,48]. Indeed, their research has shown that exposure of Delta Smelt larvae to 50 ng/L permethrin and 2 ng/L bifenthrin, for 96 h, resulted in significant hyperactivity, as well as a decrease in thigmotaxis (wall hugging). Similar observations have been reported for Zebrafish larvae exposed to permethrin at 50 μg/L for 24 h, resulting in alterations in nonmotor behavioral patterns, such as decreased defensive behaviors linked to thigmotaxis and scototaxis (dark/light preference) [44]. Direct links of these effects on survival in the field and overall fitness are difficult to predict, but these behavior alterations are likely to cause changes in ability to avoid predation, capture prey and subsequently affect reproduction success, leading to a decrease in population abundance [23,49].

The goals of the present study were to determine (1) the effect of environmentally relevant pyrethroid concentrations on Delta Smelt larvae behavior and (2) the dynamics of pyrethroid toxicity on Delta Smelt yolk-sac larvae behavior at salinities experienced by this species across their habitat range. We exposed Delta Smelt during sensitive stages of development (yolk-larvae) to low (ng/L) environmentally relevant concentrations of permethrin or bifenthrin, across a salinity gradient (0.5 to 6 PSU). Hatching and survival rates during the exposure and over six behavioral parameters were analyzed following 96 h exposures in order to assess whether salinity may alter the pyrethroids’ toxicity to Delta Smelt yolk-larvae. The behavioral alterations were studied by using a behavioral test based on photomotor response (using light and dark period) adapted for use on Delta Smelt larvae by Mundy et al. (2020) [47].

## 2. Materials and Methods

### 2.1. Larval Fish Source

In March 2020, Delta Smelt embryos were fertilized via strip spawning and maintained at 16 °C in the Delta water (0.2 PSU) [50] until 7 days post fertilization (dpf) at the UC Davis Fish Culture and Conservation Laboratory (FCCL) as approved by the University of California Davis Institutional Animal Care and Use Committee (IACUC); protocol #19841, date of approval: 20 March 2017. A total of 1920 embryos were used for two experiments. For each experiment, 960 embryos were transported at 7 dpf to our laboratory at UC Davis, and distributed in batches of 20 embryos across forty-eight, 200 mL beakers containing 100 mL of oxygenated filtered (0.2 μm) ground water at 0.5, or with supplemented with Instant Ocean^®^, at 2 or 6 PSU. Organisms were then placed in a controlled chamber at 16 ± 0.5 °C under dark conditions, where they were maintained until test initiation. Water physicochemical parameters were measured daily such as the pH (8.53–8.59, Hanna Instruments), dissolved oxygen (98%; YSI, Yellow Springs, OH, USA), salinity (0.5; 2 or 6 PSU; Hanna Instruments), ammonia (0; API, McLean, VA, USA), and temperature (16 ± 0.5 °C; Hanna Instruments, Woonsocket, RI, USA) before and after a 50% water renewal, at respective conditions.

### 2.2. Experimental Larvae Exposure

**Figure 1:**
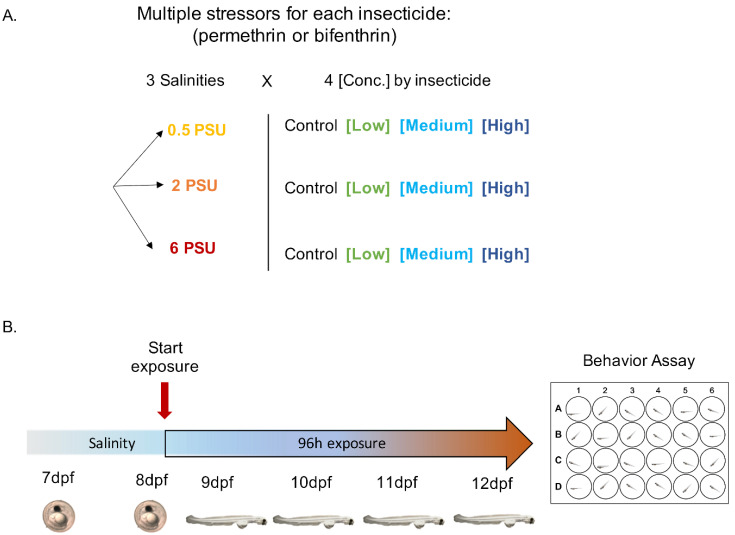
Experimental design for yolk-sac Delta Smelt larvae exposure to permethrin and bifenthrin insecticides. (**A**) Multiple stressor exposures across 3 salinities x 4 insecticide concentrations. (**B**) Exposure timeline, showing behavioral assessment conducted following 96 h exposure.

Toxicological research was approved under UC Davis IACUC protocol #20274, date of approval: 14 December 2018. Pesticide exposures were performed with either permethrin or bifenthrin, conducted using embryos from one spawn per insecticide. For each experiment, pesticide exposures were initiated at 8 dpf and included a total of 12 treatments: 3 salinities (0.5, 2 and 6 PSU) and 3 concentrations plus a solvent control (0 ng/L), in quadruplicate (Figure 1). Larvae were allowed to hatch into the exposure vessels, and were maintained under static renewal conditions for a period of 96 h. All exposures were conducted in experimental chambers at 16 ± 0.5 °C, as described above. Concentrations used in this study were chosen to reflect the concentrations measured in the San Francisco Bay Delta, California, USA [13]. The pesticide exposures were performed at 1, 10 and 100 ng/L (nominal) for permethrin (ChemService, WestChester, PA, USA. CAS: 52645-53-1, product#: N-12848), and 0.1, 1 and 10 ng/L (nominal) for bifenthrin (ChemService, WestChester, PA, USA. CAS: 82657-04-3, product#: N-11203). All concentrations (including the control) contained methanol at 0.02% *v*/*v*, used as a solvent carrier for the insecticides [51]. Water physicochemical parameters were measured daily over the 96 h exposure, at the time of 50% water renewal described above. Cumulative hatching and mortality were recorded daily. At 96 h post exposure (corresponding to 12 dpf), behavioral assays were performed on larvae from each treatment (Figure 1B).

### 2.3. Analytical Chemistry

At the beginning of each experiment, three one-liter water samples were collected from the control and the highest concentration in amber glass bottles and the effective concentrations were measured by the USGS (Sacramento, CA, USA). Bifenthrin and permethrin concentrations were measured within 24 h of sample collection using solid-phase extraction (SPE) followed by gas chromatography–mass spectrometry (GC–MS) [52]. Recoveries for each insecticide ranged from 84 to 96%. The method detection limit for 1 L of water sample is 0.5 ng/L. The actual concentrations from the highest doses were: 94.5, 92.7 or 98.6 ng/L for permethrin (nominal concentration at 100 ng/L) and 11.7, 9.3 or 10.3 ng/L for bifenthrin (nominal concentration at 10 ng/L), for 0.5, 2 and 6 PSU, respectively. No permethrin or bifenthrin was detected in the controls. We therefore report results in terms of the nominal exposure concentrations.

### 2.4. Locomotor Behavior Assay

For each experiment, behavioral tests were independently conducted at the 96 h timepoint using a 40 min Light:Dark (LD) cycle test into a DanioVision Observation Chamber (Wageningen, the Netherlands) as described by [47]. Briefly, tests were conducted in batches; a total of 10 plates (24-well cell culture plate; Thermofisher, CA, USA) were ran sequentially on a single day to evaluate behavioral responses of *n* = 20 larvae/salinity/treatment (5 larvae/replicate). Each plate held one larva from two separate replicates to ensure that the 4 replicates from each treatment were represented across the 10 plates used. Each well contained one larva and 1 mL of filtered ground water (0.22 µm) at respective salinities. Once larvae were randomly distributed across a plate, they were returned to the exposure chambers for 1 h for them to habituate to the plate before the behavioral tests were conducted. Then, each plate was carefully placed into the DanioVision Observation Chamber and after holding them for 5 min under dark conditions, the LD cycle test was started. Cycles included 10 min dark (dark 1), followed by 5 min light (light 1), 10 min dark (dark 2), 5 min light (light 2), and finally a 10 min dark period (dark 3). The resolution was set at 1280 × 960, light cycles were programmed at 10,000 lux and the frame rate was set at 25/s. During the procedure, a steady flow of water was supplied to the chamber via chiller (TECO-US, Terrell, TX, USA) to keep the fish at a constant 16 ± 0.5 °C.

### 2.5. Behavioral Parameters

Individual Delta Smelt larval activity was recorded and tracked by a Basler Gen1 Camera using the EthoVision XT 14 software (version 14, Noldus, Wageningen, the Netherlands). Video data were analyzed in 30 s time bins and several parameters were analyzed such as the total distance moved from each condition. Velocity thresholds were set experimentally and used to define the following parameters: cruising (between 0.5 to 2.0 cm/s), bursting (>2.0 cm/s), and freezing (<0.5 cm/s). Moreover, a virtual center zone (area drawn with EthoVision software as 1.3 cm diameter) was defined within each well (1.6 cm diameter) to determine the time that larvae spent in the center zone or the edge zone (well area minus center zone) across the different conditions. Z-scores were determined to increase visual clarity while presenting multiple parameters having different units (cm/s, s, %) on the same figure, and normalized to control. The calculation of Z-score was conducted using the following equation: Z-score = (x – μ)/σ, where x = value, μ= mean, σ=standard deviation.

### 2.6. Data Analysis

The homogeneity of variances and normality were tested using Levene and Shapiro–Wilk tests, respectively. Data were not normally distributed, and the variances were not homogeneous. For each experiment, we therefore analyzed changes in behavior by comparing the difference across concentrations to the controls for each salinity, using a Kruskal–Wallis test followed by Dunn’s test to determine the multiple comparison. Spearman’s correlations were performed between (1) concentration and the total distance moved for each salinity, (2) concentration and anti-thigmotaxic behavior (thigmotaxis: wall hugging; anti-thigmotaxis: time in the center zone) for each salinity, (3) salinity and the total distance moved for each concentration and (4) salinity and anti-thigmotaxic behavior for each concentration, in the dark and light cycles for both experiments (permethrin and bifenthrin independently). Only the moderate (coefficient *r_s_* = |0.3| to |0.49|) or strong (|0.5| to |1|) correlations with a significant *p* value (*, *p* < 0.05) were considered. All analyses were performed using the statistical software R (R version 3.5.1 2018, Vienna, Austria) with a significance level at *p* < 0.05. Figures were made using the statistical software R (version 3.5.1) and GraphPad Prism 8 (version 8.3.0, San Diego, CA, USA, 2019).

## 3. Results

Average hatching was 94 ± 5% and 98 ± 2% at 9 dpf, and 97 ± 2% and 98 ± 2% at 12 dpf for batches used for the permethrin and bifenthrin exposures, respectively, and there were no differences across all exposure vessels (One-way ANOVA, *p* > 0.05). Average larval survival was 97.5 ± 1.7% and 98.1 ± 1.8% for permethrin and bifenthrin experiments, respectively, across all conditions, and no difference in mortality was observed throughout the 96 h exposures. All larvae from all conditions, in both experiments, exhibited a significant increase in movement in all light periods compared to dark cycles (Figure S1), confirming predictable behavior patterns previously verified for this species [47]. No significant difference was observed between each of the three dark cycles across conditions, nor between the two light cycles (Figure S1). In addition, the total distance moved was not significantly different across concentrations, relative to controls, during the light stimulus changes, i.e., the last thirty seconds of the initial photoperiod, and the first thirty seconds of the following period (dark/light switching) for both experiments (data not shown). Therefore, the three dark cycles for each treatment were analyzed together, as were the two light cycles, facilitating the comparison between the concentrations across salinities. 

We found differences in distance moved between the controls from both experiments, which can be explained by the fact that two separate batches were used for each experiment, which is why each pesticide treatment was compared solely to their respective controls, and comparisons were not made across pyrethroids. Similar observations have already been reported between Delta Smelt batches [47,48]. This variability between batches could be due to a difference in prior early life and/or genetic diversity between the ancestors.

### 3.1. Pyrethroids Effect on Delta Smelt Larvae Behavior across A Salinity Gradient

#### 3.1.1. Permethrin

No correlation was found between the concentration and the total distance moved or the anti-thigmotaxic behavior (time spent in center zone) in both dark and light cycles (Table 1, for each salinity Spearman’s correlation coefficient *r_s_* < |0.3| and *p* > 0.05). However, significant differences were observed across concentrations and relative to controls, across a salinity gradient for multiple other behavioral endpoints under both light and dark cycles.

**Figure 2:**
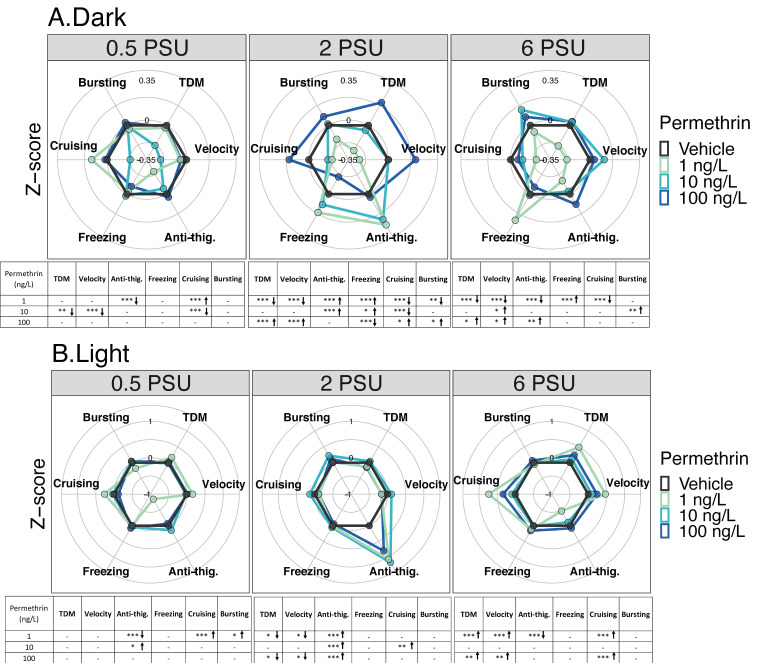
Delta Smelt yolk-sac larvae behavioral response after 96 h exposure to permethrin across a salinity gradient (0.5, 2 or 6 PSU). Mean Delta Smelt swimming activity in (**A**) dark and (**B**) light cycles periods following permethrin exposure. Plotted circles represent activity over 10 min dark (x3) or 5 min light (x2) photoperiods. Data are representative of the calculated Z-scores normalized to controls, which are presented on the 0 axis in each figure. Behavioral parameters include total distance moved (cm), velocity (cm/s), anti-thigmotaxis (or anti-thig.: time spent in the center zone), and duration of movement across three speed levels, freezing (<0.5 cm/s), cruising (0.5 to 2.0 cm/s) and bursting (>2cm/s). Arrows represent a significant increase (↑), or decrease (↓) in activity in comparison to control. *n* = 20 larvae used in behavioral observations for each treatment (insecticide concentrations /salinity). * *p* < 0.05; ** *p* < 0.005; *** *p* < 0.0005, Kruskal–Wallis test followed by Dunn’s test, comparing all concentrations to control within each cycle per salinity.

In the dark period, larvae at 0.5 PSU exposed to 1 ng/L showed a significant increase in thigmotaxic behavior, while cruising time was significantly increased compared to the control (*p* < 0.05) (Figure 2A). Under the same conditions (dark period and 0.5 PSU), hypoactivity was higher in larvae exposed to 10 ng/L than in control larvae (same salinity), while the highest concentration, 100 ng/L permethrin, showed no difference across all other behavioral endpoints. Larvae at 2 PSU exposed to 1 ng/L exhibited a significantly decrease in total distance moved, velocity, cruising duration, bursting time, and showed an increase anti-thigmotaxic behavior and freezing duration compared to the control (Figure 2A). Additionally, at 2 PSU, exposure to 10 ng/L caused an increase in anti-thigmotaxic behavior and freezing time, and overall hypoactivity in terms of the cruising duration compared to the respective control. However, larvae exposed to 100 ng/L permethrin exhibited increased movements in terms of total distance moved, velocity, cruising and bursting duration compared to the control. This resulted in a significant quadratic dose–response curve at 2 PSU, with significant hyperactivity observed in larvae exposed to 100 ng/L, during the dark cycle (Figure S2A). At 6 PSU, larvae exposed to 1 ng/L presented a significant decrease in total distance moved, velocity, and cruising duration, and an increase in thigmotaxic behavior and freezing time compared to the respective control, in the dark (Figure 2A). Larvae exposed to 10 and 100 ng/L at 6 PSU were hyperactive compared to controls. There were significant increases in velocity and bursting time at 10 ng/L, and an increase in total distance moved, velocity and anti-thigmotaxic behavior at 100 ng/L. 

In the light period, larvae at 0.5 PSU exposed to 1 ng/L showed a significant increase in thigmotaxic behavior, while cruising and bursting time were significantly increased compared to the control (*p* < 0.05) (Figure 2B). Exposure to 10 ng/L permethrin caused an increase in anti-thigmotaxis compared to the control, and larvae from the highest concentration, 100 ng/L permethrin, showed no difference across all other endpoints. Larvae at 2 PSU exposed to 1 ng/L and to 100 ng/L exhibited a significantly decrease in total distance moved, velocity, and showed an increase in anti-thigmotaxic behavior compared to the control (*p* < 0.05) (Figure 2B). Additionally, at 2 PSU, exposure to 10 ng/L caused an increase in anti-thigmotaxic behavior and well as in cruising duration compared to the control. Larvae at 6 PSU exposed to 1 ng/L and to 100 ng/L presented a significant increase in total distance moved, velocity and cruising duration (Figure 2B). An increase in thigmotaxic behavior was also determined for larvae exposed to 1 ng/L, while larvae exposed to 10 ng/L showed no difference across all other endpoints tested.

#### 3.1.2. Bifenthrin

**Figure 3:**
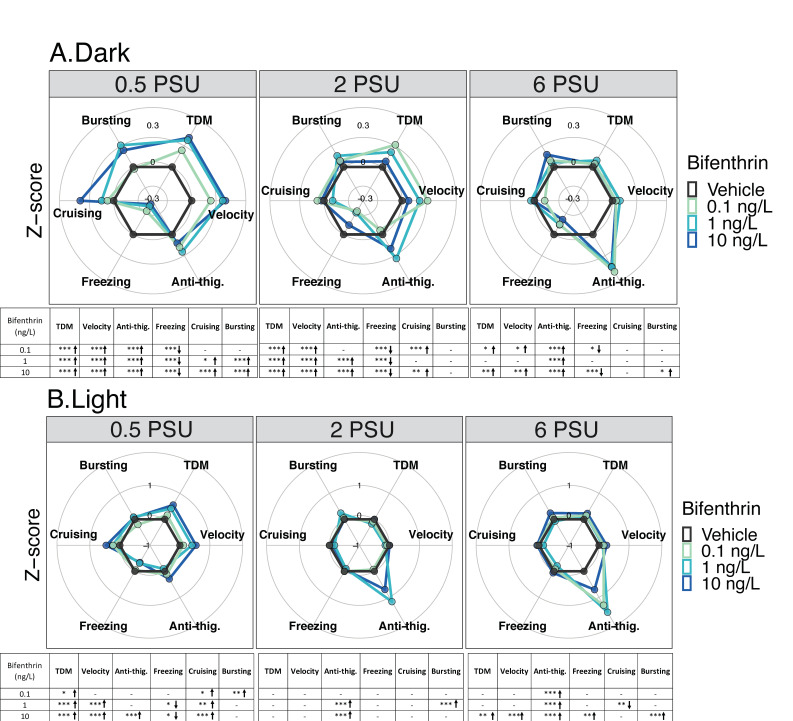
Delta Smelt yolk-sac larvae behavioral response after 96 h exposure to bifenthrin across a salinity gradient (0.5, 2 or 6 PSU). Mean Delta Smelt swimming activity in (**A**) dark and (**B**) light cycles periods following bifenthrin exposure. Plotted circles represents activity over 10 min dark (x3) or 5 min light (x2) photoperiods. Data are representative of the calculated Z-scores normalized to controls, which are presented on the 0 axis in each figure. Behavioral parameters include total distance moved (cm), velocity (cm/s), anti-thigmotaxis (or anti-thig.: time spent in the center zone), and duration of each movement across three speed levels, freezing (<0.5 cm/s), cruising (0.5 to 2.0 cm/s) and bursting (>2cm/s). Arrows represent a significant increase (↑), or decrease (↓) in activity in comparison to control. *n* = 20 larvae used in behavioral observations for each treatment (insecticide concentrations /salinity). * *p* < 0.05; ** *p* <0.005; *** *p* < 0.0005, Kruskal–Wallis test followed by Dunn’s test, comparing all concentrations to control within each cycle per salinity.

In the dark period, at 0.5 PSU, all bifenthrin tested concentrations (0.1, 1 and 10 ng/L) caused a significant increase in total distance moved, velocity, anti-thigmotaxic behavior, and a decrease in freezing duration compared to controls (Figure 3A). In addition, increases in cruising and bursting duration were also observed for larvae exposed to 1 and 10 ng/L. At 2 PSU, all bifenthrin concentrations caused an increase in total distance moved, velocity and the freezing duration decreased compared to controls. Larvae exposed to 0.1 and 10 ng/L also showed an increase in cruising duration, and those exposed to 1 and 10 ng/L spend significantly more time in the center area; increased anti-thigmotaxic behavior (Figure 3A). At 6 PSU, increased anti-thigmotaxis behavior was observed for all group exposed compared to the control groups. Larvae exposed to 0.1 and 10 ng/L displayed increased total distance moved, velocity, and decreased freezing duration compared to the control at 6 PSU (Figure 3A). 

In the light period, at 0.5 PSU, the total distance moved and cruising duration were significantly higher at all concentrations compared to controls (*p* < 0.05) (Figure 3B). The bursting duration was also significantly higher for larvae exposed to 0.1 ng/L compared to the respective control. Larvae exposed to 1 and 10 ng/L showed an increase in velocity and a decrease in freezing duration compared to the control. Only the larvae exposed to 10 ng/L spent more time in the center zone compared to the control. During the light periods, a significant dose dependent increase in the form of a linear dose response was observed at 0.5 PSU (Figure S2B). At 2 PSU, no difference was observed in terms of total distance moved between the concentrations and the control at 2 PSU in the light cycle. Nevertheless, a positive moderate correlation was observed between the concentration and the total distance moved (Table 1, *r_s_* =0.32, *p* < 0.05). At this salinity, larvae exposed to 1 and 10 ng/L spent significantly more time in the center zone compared to the control (Figure 3B) reflecting an anti-thigmotaxic behavior. In addition, a positive and moderate correlation was found between this behavioral parameter and the concentrations at this salinity (*r_s_* =0.43, *p* < 0.05) (Table 1). Bursting duration was also significantly higher for larvae exposed at 1 ng/L compared to the control. At 6 PSU, a positive but moderate correlation was found between the concentration and the anti-thigmotaxis parameter (*r_s_* = 0.33, *p* < 0.05) (Table 1). Moreover, all concentrations resulted in significant anti-thigmotaxic behavior compared to the control. Finally, under this salinity, larvae exposed to 10 ng/L showed a significant increase across all evaluated behavioral endpoints except for cruising duration compared to controls (Figure 3B).

### 3.2. Effect of the Salinity on the Toxicity of Pyrethroid Insecticides on Delta Smelt Larvae

#### 3.2.1. Permethrin

**Figure 4:**
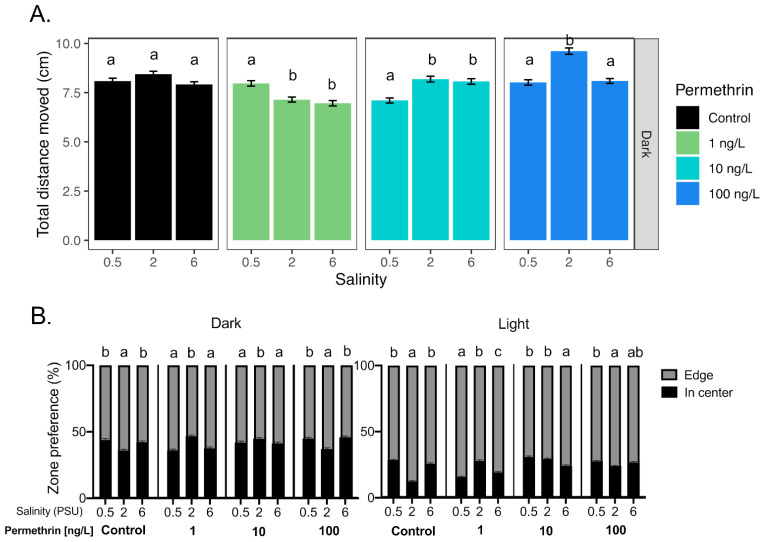
Effect of salinity on Delta Smelt yolk-sac larvae behavior exposed with permethrin. (**A**) Total distance moved response of larvae under the dark period and (**B**) thigmotaxic behavior (edge/wall preference) of Delta Smelt larvae at four permethrin concentrations (0, 1, 10 and 100 ng/L) over dark and light cycles. Letters indicate a significant difference between groups (Dunn’s test, *p* < 0.05), and error bars represent the S.E.M.

A significant effect of the salinity was observed on total distance moved from larvae exposed to all permethrin concentrations, during the dark periods, while no effect was observed in the control (Figure 4A). Indeed, at 1 and 10 ng/L of permethrin, larvae at 0.5 PSU showed a significant difference in distance moved compared to those at 2 PSU and 6 PSU (increase and decrease, respectively). At 100 ng/L, the larvae at 2 PSU showed a significant increase in movement compared to those at 0.5 and 6 PSU. No difference in the total distance moved was observed across salinities for each concentration tested during the light period (data not shown), but the thigmotaxic behavior showed significant differences across salinities in both light and dark periods (Figure 4B). However, neither the total distance moved nor the thigmotaxis were correlated with the salinity (*r_s_* < 0.3, for each concentration and periods).

#### 3.2.2. Bifenthrin

**Figure 5:**
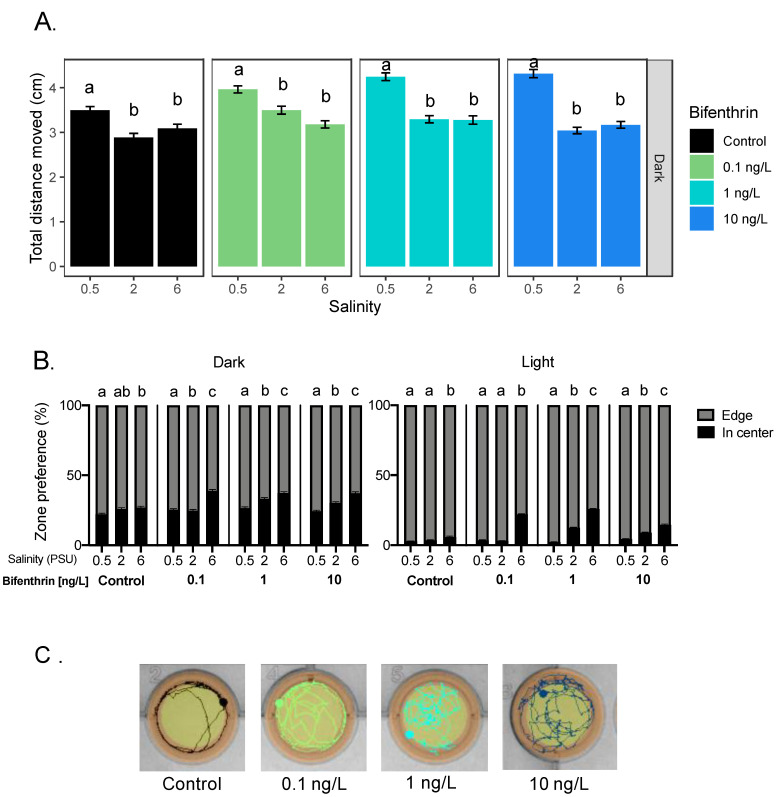
Effect of salinity on Delta Smelt yolk-sac larvae behavior exposed with bifenthrin. **(A**) Total distance moved response of larvae under the dark period and (**B**) thigmotaxic behavior (edge/wall preference) of Delta Smelt larvae at four bifenthrin concentrations (0, 0.1, 1 and 10 ng/L) over dark and light cycles, and (**C**) example locomotion trace of individual larval Delta Smelt performing thigmotaxis (wall/edge preference) in the control, to a lesser extent, in the exposed larvae. (orange: edge zone; yellow: in center zone of the well) in the dark period at 6 PSU. In the bar graphs, different letters indicate a significant difference between groups (Dunn’s test, *p* < 0.05), and error bars represent the S.E.M.

All tested bifenthrin concentrations, including the control, caused a significant increase in total distance moved at 0.5 PSU compared to 2 and 6 PSU during the dark period (Figure 5A). Anti-thigmotaxic behavior, however, significantly increased with increasing salinity and bifenthrin concentrations during in both light and dark periods (Figure 5B,C). Positive correlations were observed in the light period, between anti-thigmotaxic behavior and salinity at each of the tested bifenthrin concentrations (0.1, 1 and 10 ng/L; *r_s_* = 0.67, 0.83 and 0.46, respectively, *p* < 0.05) (Table 1).

## 4. Discussion

Our data describe strong patterns of hypoactivity for larvae exposed to permethrin at low concentrations and low salinity, while those exposed at high concentrations and high salinity exhibited hyperactivity. Bifenthrin, on the other hand, caused hyperactivity at all concentrations and salinities compared to controls, and thigmotaxis (wall hugging) negatively correlated with salinity. These results suggest that (1) environmentally relevant pyrethroid concentrations can perturb larval Delta Smelt behavior and may alter larval anxiety levels, even at concentrations assumed to safe for aquatic species, and (2) salinity can change the dynamic of pyrethroid toxicity in terms of behavioral effects on Delta Smelt.

### 4.1. Low Pyrethroid Concentrations Impact Early Larval Delta Smelt Behavior and Decrease Their Anxiety-Related Response

In the present study, we tested concentrations of pyrethroid insecticides that are frequently detected at locations where Delta Smelt spawn and during their spawning season. We have shown that a short-term exposure (96 h) to concentrations as low as 1 and 0.1 ng/L permethrin and bifenthrin, respectively, can impact behavior. When we compared the locomotor behavior between the conditions (exposed versus control across a salinity gradient), multiple endpoints (distance moved, velocity, freezing, cruising, bursting and thigmotaxis) confirmed that yolk-sac larvae exposed to permethrin were either hypo-or hyperactive depending on concentration and salinity, while larvae exposed to bifenthrin exhibited hyperactivity across both concentration and salinity, interactively.

To date, understanding Delta Smelt behavior and its response to insecticide exposure is limited to a few studies [47,48,53]. Several hypotheses can explain behavioral changes associated with sublethal exposure. Among them, an alteration of genes involved in neurodevelopment due to exposure can result in a locomotion alteration. Investigating larval behavior and associated molecular change after pyrethroid exposure, can provide important clues toward identifying the neural mechanisms involved [45,53], but clear mechanisms are still unknown. Mundy et al. (2020) has demonstrated that environmentally relevant concentrations of bifenthrin that caused hyperactivity (at 2, 10 and 100 ng/L) may impact genes involved in neurodevelopment (at 100 ng/L) of 12 days post hatched Delta Smelt larvae [47]. Indeed, the same authors suggested that mechanisms other than neuronal injury may be responsible for resulting hyperactivity at lower concentrations of bifenthrin (2 and 10 ng/L), pointing to potential long-term effects of exposure, through impacts on neurodevelopment. The range of concentrations used in our study includes concentrations that are much lower than maximum concentrations detected in tributaries to their habitat during a storm event (permethrin 66.1 ng/L and bifenthrin up to 106 ng/L [54,55]) and likely encompass concentrations that are representative of chronic and acute exposures.

Among the different behavioral endpoints measured in this study, thigmotaxis (also known as wall hugging) showed a strong pattern for both experiments, with a result of negative thigmotaxis in exposed larvae compared to the control across the salinity gradient. Larvae exposed to bifenthrin were spending more time in the center compared to the control at all salinities; for larvae exposed to permethrin, negative thigmotaxis, especially at 2 PSU, was observed at all concentrations. Previous studies also reported a negative thigmotaxis in Delta Smelt larvae exposed to permethrin and bifenthrin [47,48]. This parameter is usually taken as a measure of anxiety or fear levels and evolutionarily conserved in different species such as rodents, fish and humans [56]. Indeed, in thigmotaxis, the animal avoids the center of the arena and moves in contact with a vertical surface (wall for example), especially in a novel environment, which indicates some form of anxiety. This endpoint is crucial in studying anxiogenic (such as caffeine and pentylenetetrazol) and anxiolytic/sedative (such as diazepam) drugs. Thus, results of the present study suggested that Delta Smelt larvae exposed to sublethal concentrations of pyrethroid insecticides, equal to or lower than 100 ng/L, present less anxiety-related behavior when they are presented with a visual stimulus. An increase in the amount of time in the center or open zone, depending on the test, is considered to be reflective of an anxiolytic response to a compound such as caffeine [57]. Nunes et al. (2019) also advanced the hypothesis that permethrin exposure (50 μg/L) may have decreased the anxiety-like behavior in Zebrafish larvae, altering the normal preference of the periphery in favor of the central area [44]. This change of anxiety related behavior after a pyrethroid exposure may be a threat to the survival of these larvae in terms of predator avoidance and food capture. Moreover, Frank et al. (2019) demonstrated that bifenthrin can alter predator avoidance behavior in Inland Silversides larvae exposed at 3 and 27 ng/L [46]. Anxiety is also an important factor in assessment of an animal’s spatial learning ability since it can impair spatial learning and memory [58,59]. Indeed, early life permethrin exposure in rats has shown long-lasting consequences on the hippocampus such as impairment of long-term memory storage, resulting in spatial learning deficits [60]. Moreover, it has previously been reported that the pyrethroid increased incidence of eye cataracts in adult Nile tilapia, suggesting the possibility that bifenthrin-induced behavioral deficits in response to light-dark stimuli are secondary to ocular toxicity [61]. This information raises the hypothesis that (1) pyrethroids inhibited fear (anxiolytic proprieties) on Delta Smelt larvae, resulting in motivation to explore the environment, and/or (2) these insecticides impair spatial perception in larvae. However, our data indicate that while exposed larvae spend more time in the center of the well compare to controls (at each salinity), the larvae are still able to detect the visual stimuli and respond to these dark and light signals by exhibiting a clear preference to swim during a visual stimulus (light versus dark cycles) (Figure S1). The anti-thigmotaxis and bifenthrin concentrations were positively correlated at 2 and 6 PSU, and larvae exhibited increase in bursting and freezing at 6 PSU at the highest concentration suggesting an erratic behavior and a motor deficit when exposed at high concentration and salinity.

### 4.2. Salinity Increases Pyrethroid Toxicity, Resulting in Behavior Change

Salinity changes occurring due to global climate change may enhance pesticides toxicity effects in aquatic organisms by altering their chemical fate and transport [62,63,64,65,66]. Salinity can interact in a synergistic, additive or antagonistic manner with pesticides, thereby altering the tolerance of the animal [67]. Understanding how salinity can affect the toxicity of pyrethroids is crucial to early life stages of fishes, which are at high risk of exposure, especially during a storm runoff event when pesticide concentrations increase rapidly. The influence of salinity on toxicity tolerance has been reported in other species including shrimps, amphipods and fish [68,69,70], and several pesticides seem to be more toxic at high salinities [71,72]. Bifenthrin is potentially more toxic for aquatic organisms at higher salinity [64,69]. For example, in the amphipod *Hyalella azteca*, the combination of an increase in bifenthrin toxicity at low temperature, with increasing salinity, reduced the organisms’ capability to contend with nanomolar exposures (1 ng/L) [69]. In our study, salinity was found to be positively correlated with anti-thigmotaxic behavior at each bifenthrin concentration, while no correlation was observed for permethrin exposed larvae. This suggests that bifenthrin may be more toxic at high salinities, resulting in an alteration of fish locomotion. Several mechanisms can explain that the salinity enhances the toxicity of organic compound, including (1) salinity may inhibit detoxification pathways, enhance bioactivation pathways, or reduce elimination of the compounds, (2) salinity may increase the uptake and bioaccumulation of xenobiotics, (3) salinity may induce a chemical change of the compounds and/or (4) larval osmoregulation may be altered by pesticides at high salinity. The lipophilic properties of pyrethroids, its permeation into adipose tissues, skin, ovaries, kidneys, adrenal glands, and liver of aquatic animal is relatively high [18] and results in elevated levels of bioaccumulation in those tissues. Early life stages such as yolk-sac larvae, thus may be the most vulnerable to these pesticides due to the yolk nature which is highly adipose.

The hydrophobicity of pyrethroids also plays a role in its affinity for tissues (solid phase) at high salinity [35]. However, hydrophobicity alone cannot explain that bifenthrin toxicity was correlated with the salinity and permethrin was not, since both are hydrophobic and have similar water solubility coefficients (octanol–water partition coefficient or *K*_ow_ = 6.9 and 6.4 for permethrin and bifenthrin, respectively) [73]. The different interactions observed between salinity and these insecticides may be explained by a difference in mechanism of action. While both are type I pyrethroids because they lack an α-CN group present in type II pyrethroids, previous studies suggest a mixed type I/II mode of action for the bifenthrin [74]. The mechanism of action of type I pyrethroids is to change the sodium channels’ conformation during their opening and closing in neuronal membranes [19,75,76]. The channel opening duration is dependent on the pyrethroid type, with type II pyrethroids holding voltage-gated sodium channels open much longer than type I [77]. Moreover, pyrethroids can inhibit the Ca^2+^channels and the Ca^2+^/Mg^2+^ ATPases. This alteration is considered a secondary toxic mechanism of pyrethroids related to osmoregulation disorders because these ATPases are involved in ion regulation [78]. More studies are needed to investigate the different mode of action between both pyrethroids and their interactions with salinity. Linking larvae behavior and molecular change will give important clues to identify the neuronal mechanisms involved across a salinity gradient.

## 5. Conclusions

Our study demonstrated that low, environmentally relevant, concentrations of pyrethroids impact larval Delta Smelt behavior, which may have long-term consequences. This study also confirms that increases in salinity can increase pyrethroid toxicity on exposed larvae, especially for bifenthrin. Understanding the biological consequences of interactions between salinity and contaminants in the highly managed San Francisco Bay Delta is particularly important because alternate management actions can strongly influence abiotic conditions and affect Delta Smelt habitat, its fitness, population, and potential for recovery. These findings also have far-reaching implications across coastal and estuarine ecosystems, given that pesticides and other hydrophobic contaminants present in runoff and effluent could be more severely impacting sensitive fish species in saline habitats in comparison to freshwater. As such, it is critical that risk assessments consider the role of abiotic factors, such as salinity, to facilitate the protection of fishes globally as our climate continues to change.

## Figures and Tables

**Table 1:** Correlation between Delta Smelt yolk-sac behavioral parameters (either the total distance moved or the anti-thigmotaxic behavior) and the insecticide concentration, or the salinity for each treatment in the dark and light period, for both permethrin and bifenthrin exposures. TDM: total distance moved. * *p* < 0.05, and *r_s_* is the Spearman’s correlation coefficient. Only the moderate (|0.3| to |0.49|) or strong (|0.5| to |1|) correlations with a * *p <* 0.05 were considered. These correlations were highlighted in orange (moderate) or red (strong). ns: not significant.

Correlation (Spearman)		Condition	Periods			
			Dark		Light	
			*r_s_*	*p*	*r_s_*	*p*
**Permethrin**	Concentration vs. TDM	0.5 PSU	0.00	ns	−0.02	ns
		2 PSU	0.09	*	−0.04	ns
		6 PSU	0.06	*	0.02	ns
	Concentration vs. anti-thigmotaxis	0.5 PSU	0.11	*	0.21	*
		2 PSU	0.05	*	−0.01	ns
		6 PSU	0.05	*	0.08	*
	Salinity vs. TDM	Control	0.01	ns	−0.05	ns
		1 ng/L	−0.09	*	0.08	*
		10 ng/L	0.09	*	−0.06	ns
		100 ng/L	0.03	ns	0.04	ns
	Salinity vs. anti-thigmotaxis	Control	−0.14	ns	−0.01	ns
		1 ng/L	−0.19	*	−0.02	ns
		10 ng/L	−0.24	*	−0.22	*
		100 ng/L	−0.28	*	−0.16	*
**Bifenthrin**	Concentration vs. TDM	0.5 PSU	0.04	*	0.10	*
		2 PSU	0.00	ns	**0.32**	*
		6 PSU	0.05	*	0.07	*
	Concentration vs. anti-thigmotaxis	0.5 PSU	0.07	*	0.18	*
		2 PSU	0.09	*	**0.43**	*
		6 PSU	0.11	*	**0.33**	*
	Salinity vs. TDM	Control	−0.02	ns	−0.10	*
		0.1 ng/L	0.00	ns	0.13	*
		1 ng/L	−0.01	ns	−0.18	*
		10 ng/L	0.01	ns	−0.02	ns
	Salinity vs. anti-thigmotaxis	Control	0.05	*	0.16	*
		0.1 ng/L	0.14	*	**0.67**	*
		1 ng/L	0.10	*	**0.83**	*
		10 ng/L	0.14	*	**0.46**	*

## Data Availability

The data presented in this study are available on request from the corresponding author.

## Supplementary Materials

The following are available online at https://www.mdpi.com/2305-6304/9/2/40/s1, Figure S1: Total distance moved by Delta Smelt yolk-sac larvae after 96 h exposure to pyrethroids under each dark and light cycle, across a salinity gradient (0.5, 2 or 6 PSU). Figure S2: Dose responses within dark/light cycle across a salinity gradient (0.5, 2 and 6 PSU): (A) Delta Smelt larvae exposed to 0 (controls), 1, 10 and 100 ng/L permethrin, (B) Delta Smelt larvae exposed to 0 (controls), 0.1, 1 and 10 ng/L bifenthrin. Each dot represents the mean total distance moved of one individual, *n* = 20 larvae/treatment. Data were rescaled between 0 and 1 to facilitate comparison between periods, as well as pesticides. Five dose–response curves were fit using a maximum likelihood approach: linear, quadratic, sigmoidal, unimodal1, and unimodal2. Curves shown as a solid line are significantly better fits than a null intercept-only model (*p* < 0.05), curves shown as a dashed line are the best fit of the five-curve option (lowest *p*-value), but not significantly better than the null model.

## References

[1] Hobbs J.A., Lewis L.S., Willmes M., Denney C., Bush E. (2019). Complex Life Histories Discovered in a Critically Endangered Fish. Sci. Rep..

[2] Bennett W.A. (2005). Critical Assessment of the Delta Smelt Population in the San Francisco Estuary, California. San Franc. Estuary Watershed Sci..

[3] Moyle P.B., Herbold B., Stevens D.E., Miller L.W. (1992). Life History and Status of Delta Smelt in the Sacramento-San Joaquin Estuary, California. Trans. Am. Fish. Soc..

[4] California Department of Fish and Wildlife (CDFW) (2014). State & Federally Listed Endangered & Threatened Animals of California, Department of Fish and Wildlife, Bio-Geographic Data Branch.

[5] IUCN (2014). Hypomesus Transpacificus: NatureServe: The IUCN Red List of Threatened Species.

[6] Sommer T., Armor C., Baxter R., Breuer R., Brown L., Chotkowski M., Culberson S., Feyrer F., Gingras M., Herbold B. (2007). The Collapse of Pelagic Fishes in the Upper San Francisco Estuary: El Colapso de Los Peces Pelagicos En La Cabecera Del Estuario San Francisco. Fisheries.

[7] Kuivila K.M., Moon G.E. (2004). Potential Exposure of Larval and Juvenile Delta Smelt to Dissolved Pesticides in the Sacramento-San Joaquin Delta, California. Am. Fish. Soc. Symp..

[8] Brooks M.L., Fleishman E., Brown L.R., Lehman P.W., Werner I., Scholz N., Mitchelmore C., Lovvorn J.R., Johnson M.L., Schlenk D. (2012). Life Histories, Salinity Zones, and Sublethal Contributions of Contaminants to Pelagic Fish Declines Illustrated with a Case Study of San Francisco Estuary, California, USA. Estuaries Coasts.

[9] Moyle P., Brown L., Durand J., Hobbs J. (2016). Delta Smelt: Life History and Decline of a Once-Abundant Species in the San Francisco Estuary. SFEWS.

[10] Fong S., Louie S., Werner I., Connon R.E. (2016). Contaminant Effects on California Bay–Delta Species and Human Health. SFEWS.

[11] Connon R., Hasenbein S., Brander S., Poynton H., Holland E., Schlenk D., Orlando J., Hladik M., Collier T., Scholz N. (2019). Review of and Recommendations for Monitoring Contaminants and Their Effects in the San Francisco Bay−Delta. SFEWS.

[12] Weston D., Moschet C., Young T., Johanif N., Poynton H., Major K., Connon R., Hasenbein S. (2019). Chemical and Toxicological Impacts to Cache Slough Following Storm-Driven Contaminant Inputs. SFEWS.

[13] Weston D.P., Lydy M.J. (2012). Stormwater Input of Pyrethroid Insecticides to an Urban River. Environ. Toxicol. Chem..

[14] Weston D.P., Schlenk D., Riar N., Lydy M.J., Brooks M.L. (2015). Effects of Pyrethroid Insecticides in Urban Runoff on Chinook Salmon, Steelhead Trout, and Their Invertebrate Prey. Environ. Toxicol. Chem..

[15] Weston D.P., Asbell A.M., Lesmeister S.A., Teh S.J., Lydy M.J. (2014). Urban and Agricultural Pesticide Inputs to a Critical Habitat for the Threatened Delta Smelt (Hypomesus Transpacificus). Environ. Toxicol. Chem..

[16] Deanovic L.A., Stillway M., Hammock B.G., Fong S., Werner I. (2018). Tracking Pyrethroid Toxicity in Surface Water Samples: Exposure Dynamics and Toxicity Identification Tools for Laboratory Tests with Hyalella Azteca (Amphipoda). Environ. Toxicol. Chem..

[17] Deng X. (2019). Study 321. Surface Water Monitoring for Pesticides in Agricultural Areas in Central Coast and Southern California.

[18] Costa C., Rapisarda V., Catania S., Di Nola C., Ledda C., Fenga C. (2013). Cytokine Patterns in Greenhouse Workers Occupationally Exposed to α-Cypermethrin: An Observational Study. Environ. Toxicol. Pharm..

[19] Soderlund D.M. (2012). Molecular Mechanisms of Pyrethroid Insecticide Neurotoxicity: Recent Advances. Arch. Toxicol..

[20] Brander S.M., Gabler M.K., Fowler N.L., Connon R.E., Schlenk D. (2016). Pyrethroid Pesticides as Endocrine Disruptors: Molecular Mechanisms in Vertebrates with a Focus on Fishes. Environ. Sci. Technol..

[21] Clark J.M., Matsumura F. (1982). Two Different Types of Inhibitory Effects of Pyrethroids on Nerve Ca- and Ca + Mg-ATPase Activity in the Squid, Loligo Pealei. Pestic. Biochem. Physiol..

[22] Singh P.B., Singh V. (2008). Cypermethrin Induced Histological Changes in Gonadotrophic Cells, Liver, Gonads, Plasma Levels of Estradiol-17β and 11-Ketotestosterone, and Sperm Motility in Heteropneustes Fossilis (Bloch). Chemosphere.

[23] Werner I., Moran K. (2008). Effects of Pyrethroid Insecticides on Aquatic Organisms. Synthetic Pyrethroids.

[24] Brander S.M., He G., Smalling K.L., Denison M.S., Cherr G.N. (2012). The in Vivo Estrogenic and in Vitro Anti-Estrogenic Activity of Permethrin and Bifenthrin. Environ. Toxicol. Chem..

[25] Brander S.M., Jeffries K.M., Cole B.J., DeCourten B.M., White J.W., Hasenbein S., Fangue N.A., Connon R.E. (2016). Transcriptomic Changes Underlie Altered Egg Protein Production and Reduced Fecundity in an Estuarine Model Fish Exposed to Bifenthrin. Aquat. Toxicol..

[26] Tyler C.R., Beresford N., van der Woning M., Sumpter J.P., Tchorpe K. (2000). Metabolism and Environmental Degradation of Pyrethroid Insecticides Produce Compounds with Endocrine Activities. Environ. Toxicol. Chem..

[27] DeGroot B.C., Brander S.M. (2014). The Role of P450 Metabolism in the Estrogenic Activity of Bifenthrin in Fish. Aquat. Toxicol..

[28] Nillos M.G., Chajkowski S., Rimoldi J.M., Gan J., Lavado R., Schlenk D. (2010). Stereoselective Biotransformation of Permethrin to Estrogenic Metabolites in Fish. Chem. Res. Toxicol..

[29] DeCourten B.M., Brander S.M. (2017). Combined Effects of Increased Temperature and Endocrine Disrupting Pollutants on Sex Determination, Survival, and Development across Generations. Sci. Rep..

[30] Duffy T.A., McElroy A.E., Conover D.O. (2009). Variable Susceptibility and Response to Estrogenic Chemicals in Menidia Menidia. Mar. Ecol. Prog. Ser..

[31] Kidd K.A., Blanchfield P.J., Mills K.H., Palace V.P., Evans R.E., Lazorchak J.M., Flick R.W. (2007). Collapse of a Fish Population after Exposure to a Synthetic Estrogen. Proc. Natl. Acad. Sci. USA.

[32] Kimmerer W.J., MacWilliams M., Gross E.S. (2013). Variation of Fish Habitat and Extent of the Low-Salinity Zone with Freshwater Flow in the San Francisco Estuary. SFEWS.

[33] Brown L.R., Bennett W.A., Wagner R.W., Morgan-King T., Knowles N., Feyrer F., Schoellhamer D.H., Stacey M.T., Dettinger M. (2013). Implications for Future Survival of Delta Smelt from Four Climate Change Scenarios for the Sacramento–San Joaquin Delta, California. Estuaries Coasts.

[34] Saranjampour P., Vebrosky E.N., Armbrust K.L. (2017). Salinity Impacts on Water Solubility and N-Octanol/Water Partition Coefficients of Selected Pesticides and Oil Constituents. Environ. Toxicol. Chem..

[35] Yang L., Cheng Q., Lin L., Wang X., Chen B., Luan T., Tam N.F.Y. (2016). Partitions and Vertical Profiles of 9 Endocrine Disrupting Chemicals in an Estuarine Environment: Effect of Tide, Particle Size and Salinity. Environ. Pollut..

[36] Hladik M.L. (2020). Partitioning of Six Pyrethroid Insecticides at Varying Salinities: U.S. Geological Survey Data Release.

[37] Basnet R.M., Zizioli D., Taweedet S., Finazzi D., Memo M. (2019). Zebrafish Larvae as a Behavioral Model in Neuropharmacology. Biomedicines.

[38] Fraser T.W.K., Khezri A., Jusdado J.G.H., Lewandowska-Sabat A.M., Henry T., Ropstad E. (2017). Toxicant Induced Behavioural Aberrations in Larval Zebrafish Are Dependent on Minor Methodological Alterations. Toxicol. Lett..

[39] Zhang B., Xu T., Huang G., Yin D., Zhang Q., Yang X. (2018). Neurobehavioral Effects of Two Metabolites of BDE-47 (6-OH-BDE-47 and 6-MeO-BDE-47) on Zebrafish Larvae. Chemosphere.

[40] Ellis L.D., Seibert J., Soanes K.H. (2012). Distinct Models of Induced Hyperactivity in Zebrafish Larvae. Brain Res..

[41] Irons T.D., MacPhail R.C., Hunter D.L., Padilla S. (2010). Acute Neuroactive Drug Exposures Alter Locomotor Activity in Larval Zebrafish. Neurotoxicol. Teratol..

[42] Bandara S.B., Carty D.R., Singh V., Harvey D.J., Vasylieva N., Pressly B., Wulff H., Lein P.J. (2020). Susceptibility of Larval Zebrafish to the Seizurogenic Activity of GABA Type A Receptor Antagonists. NeuroToxicology.

[43] DeMicco A., Cooper K.R., Richardson J.R., White L.A. (2010). Developmental Neurotoxicity of Pyrethroid Insecticides in Zebrafish Embryos. Toxicol. Sci..

[44] Nunes M.E.M., Schimith L.E., da Costa-Silva D.G., Lopes A.R., Leandro L.P., Martins I.K., de Mello R.S., Hartmann D.D., de Carvalho N.R., da Rosa P.C. (2019). Acute Exposure to Permethrin Modulates Behavioral Functions, Redox, and Bioenergetics Parameters and Induces DNA Damage and Cell Death in Larval Zebrafish. Oxidative Med. Cell. Longev..

[45] Frank D.F., Miller G.W., Harvey D.J., Brander S.M., Geist J., Connon R.E., Lein P.J. (2018). Bifenthrin Causes Transcriptomic Alterations in MTOR and Ryanodine Receptor-Dependent Signaling and Delayed Hyperactivity in Developing Zebrafish (Danio Rerio). Aquat. Toxicol..

[46] Frank D.F., Brander S.M., Hasenbein S., Harvey D.J., Lein P.J., Geist J., Connon R.E. (2019). Developmental Exposure to Environmentally Relevant Concentrations of Bifenthrin Alters Transcription of MTOR and Ryanodine Receptor-Dependent Signaling Molecules and Impairs Predator Avoidance Behavior across Early Life Stages in Inland Silversides (Menidia Beryllina). Aquat. Toxicol..

[47] Mundy P.C., Carte M.F., Brander S.M., Hung T.-C., Fangue N., Connon R.E. (2020). Bifenthrin Exposure Causes Hyperactivity in Early Larval Stages of an Endangered Fish Species at Concentrations That Occur during Their Hatching Season. Aquat. Toxicol..

[48] Mundy P., Huff Hartz K., Fulton C., Lydy M., Brander S., Hung T., Fangue N., Connon R. (2020). Exposure to Permethrin or Chlorpyrifos Causes Differential Dose- and Time-Dependent Behavioral Effects at Early Larval Stages of an Endangered Teleost Species. Endang. Species Res..

[49] Weis J., Candelmo A. (2012). Pollutants and Fish Predator/Prey Behavior: A Review of Laboratory and Field Approaches. Curr. Zool..

[50] Baskerville-Bridges B., Lindberg J.C., Doroshov S.I. (2005). Manual for the Intensive Culture of Delta Smelt (Hypomesus Transpacificus).

[51] (2014). ASTM E1192-97 Guide for Conducting Acute Toxicity Tests on Aqueous Ambient Samples and Effluents with Fishes, Macroinvertebrates, and Amphibians.

[52] Hladik M., Smalling K.L., Kuivila K. (2009). Methods of Analysis: Determination of Pyrethroid Insecticides in Water and Sediment Using Gas Chromatography/Mass Spectrometry.

[53] Connon R.E., Geist J., Pfeiff J., Loguinov A.V., D’Abronzo L.S., Wintz H., Vulpe C.D., Werner I. (2009). Linking Mechanistic and Behavioral Responses to Sublethal Esfenvalerate Exposure in the Endangered Delta Smelt; Hypomesus Transpacificus (Fam. Osmeridae). BMC Genom..

[54] Deng X. (2017). Study 304. Surface Water Monitoring for Pesticides in Agricultural Areas in Central Coast and Southern California.

[55] Weston D.P., Holmes R.W., Lydy M.J. (2009). Residential Runoff as a Source of Pyrethroid Pesticides to Urban Creeks. Environ. Pollut..

[56] Simon P., Dupuis R., Costentin J. (1994). Thigmotaxis as an Index of Anxiety in Mice: Influence of Dopaminergic Transmissions. Behav. Brain Res..

[57] Baldwin H.A., Johnston A.L., File S.E. (1989). Antagonistic Effects of Caffeine and Yohimbine in Animal Tests of Anxiety. Eur. J. Pharm..

[58] Goodman J., McIntyre C.K. (2017). Impaired Spatial Memory and Enhanced Habit Memory in a Rat Model of Post-Traumatic Stress Disorder. Front. Pharmacol..

[59] Packard M.G., Wingard J.C. (2004). Amygdala and “Emotional” Modulation of the Relative Use of Multiple Memory Systems. Neurobiol. Learn. Mem..

[60] Nasuti C., Fattoretti P., Carloni M., Fedeli D., Ubaldi M., Ciccocioppo R., Gabbianelli R. (2014). Neonatal Exposure to Permethrin Pesticide Causes Lifelong Fear and Spatial Learning Deficits and Alters Hippocampal Morphology of Synapses. J. Neurodev. Disord..

[61] El-Sayed Y.S., Saad T.T. (2008). Subacute Intoxication of a Deltamethrin-Based Preparation (Butox^®^ 5% EC) in Monosex Nile Tilapia, Oreochromis Niloticus L.. Basic Clin. Pharmacol. Toxicol..

[62] Tu H.T., Silvestre F., Meulder B.D., Thome J.-P., Phuong N.T., Kestemont P. (2012). Combined Effects of Deltamethrin, Temperature and Salinity on Oxidative Stress Biomarkers and Acetylcholinesterase Activity in the Black Tiger Shrimp (Penaeus Monodon). Chemosphere.

[63] Lin Y.-C., Chen J.-C. (2003). Acute Toxicity of Nitrite on Litopenaeus Vannamei (Boone) Juveniles at Different Salinity Levels. Aquaculture.

[64] DeLorenzo M.E., Wallace S.C., Danese L.E., Baird T.D. (2009). Temperature and Salinity Effects on the Toxicity of Common Pesticides to the Grass Shrimp, Palaemonetes Pugio. J. Environ. Sci. Health B.

[65] DeLorenzo M.E., Danese L.E., Baird T.D. (2013). Influence of Increasing Temperature and Salinity on Herbicide Toxicity in Estuarine Phytoplankton. Environ. Toxicol..

[66] DeCourten B., Romney A., Brander S., Ahuja S. (2019). Chapter 2—The Heat Is On: Complexities of Aquatic Endocrine Disruption in a Changing Global Climate. Separation Science and Technology.

[67] Velasco J., Gutiérrez-Cánovas C., Botella-Cruz M., Sánchez-Fernández D., Arribas P., Carbonell J.A., Millán A., Pallarés S. (2019). Effects of Salinity Changes on Aquatic Organisms in a Multiple Stressor Context. Philos. Trans. R. Soc. B Biol. Sci..

[68] Staton J.L., Schizas N.V., Klosterhaus S.L., Griffitt R.J., Chandler G.T., Coull B.C. (2002). Effect of Salinity Variation and Pesticide Exposure on an Estuarine Harpacticoid Copepod, Microarthridion Littorale (Poppe), in the Southeastern US. J. Exp. Mar. Biol. Ecol..

[69] Hasenbein S., Poynton H., Connon R.E. (2018). Contaminant Exposure Effects in a Changing Climate: How Multiple Stressors Can Multiply Exposure Effects in the Amphipod Hyalella Azteca. Ecotoxicology.

[70] Wang J., Grisle S., Schlenk D. (2001). Effects of Salinity on Aldicarb Toxicity in Juvenile Rainbow Trout (Oncorhynchus Mykiss) and Striped Bass (Morone Saxatilis × Chrysops). Toxicol. Sci..

[71] Brecken-Folse J.A., Mayer F.L., Pedigo L.E., Marking L.L. (1994). Acute Toxicity of 4-Nitrophenol, 2,4-Dinitrophenol, Terbufos and Trichlorfon to Grass Shrimp (Palaemonetes Spp.) and Sheepshead Minnows (Cyprinodon Variegatus) as Affected by Salinity and Temperature. Environ. Toxicol. Chem..

[72] Hall L.W., Ziegenfuss M.C., Anderson R.D., Spittler T.D., Leichtweis H.C. (1994). Influence of Salinity on Atrazine Toxicity to a Chesapeake Bay Copepod (Eurytemora Affinis) and Fish (Cyprinodon Variegatus). Estuaries.

[73] Laskowski D.A. (2002). Physical and Chemical Properties of Pyrethroids. Rev. Environ. Contam. Toxicol..

[74] Gammon D.W., Liu Z., Chandrasekaran A., El-Naggar S.F., Kuryshev Y.A., Jackson S. (2019). Pyrethroid Neurotoxicity Studies with Bifenthrin Indicate a Mixed Type I/II Mode of Action. Pest Manag. Sci..

[75] Bradberry S.M., Cage S.A., Proudfoot A.T., Vale J.A. (2005). Poisoning Due to Pyrethroids. Toxicol. Rev..

[76] Costa L.G. (2015). The Neurotoxicity of Organochlorine and Pyrethroid Pesticides. Handb. Clin. Neurol..

[77] Wakeling E.N., Neal A., Atchison W. (2012). Pyrethroids and Their Effects on Ion Channels. Pesticides—Advances in Chemical and Botanical Pesticides.

[78] Vieira C.E.D., dos Reis Martinez C.B. (2018). The Pyrethroid λ-Cyhalothrin Induces Biochemical, Genotoxic, and Physiological Alterations in the Teleost Prochilodus Lineatus. Chemosphere.

