# Supplementary material for: Salinity Changes the Dynamics of Pyrethroid Toxicity in Terms of Behavioral Effects on Newly Hatched Delta Smelt Larvae
Source: Toxics. 2021 Feb 20;9(2):40. doi: 10.3390/toxics9020040 (PMC7924609; doi:10.3390/toxics9020040)
Supplement: Supplementary file 1 [file toxics-09-00040-s001.pdf]

Supplementary Materials: Salinity Changes the Dynamics of Pyrethroid Toxicity in Terms of Behavioral Effects on Newly Hatched Delta Smelt Larvae

Amelie Segarra, Florian Mauduit, Nermeen R. Amer, Felix Biefel, Michelle L. Hladik, Richard E. Connon and Susanne M. Brander

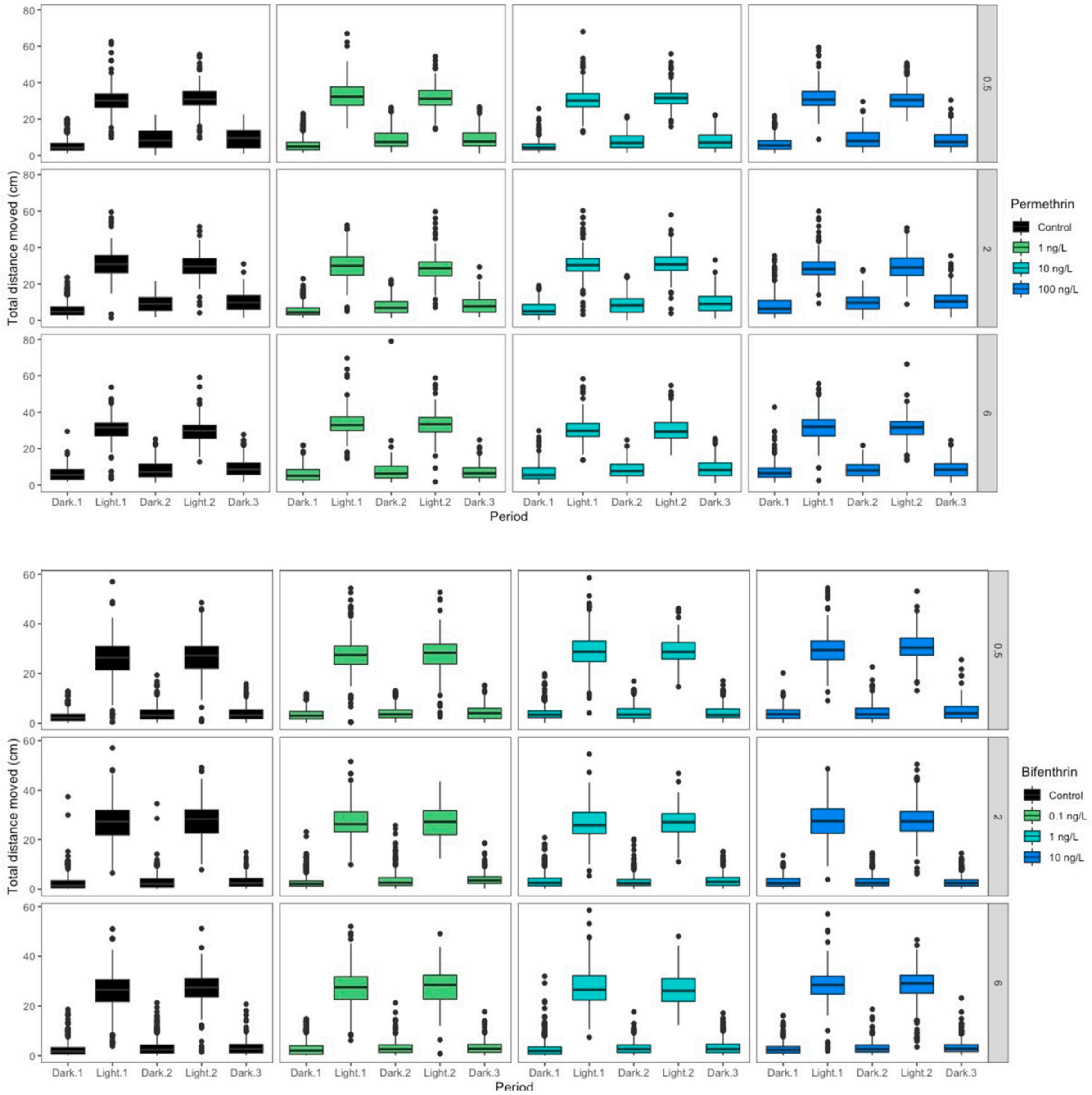

Figure S1. Total distance moved by Delta Smelt yolk-sac larvae after 96 h exposure to pyrethroids under each dark and light cycles, across a salinity gradient (0.5, 2 or 6 PSU).

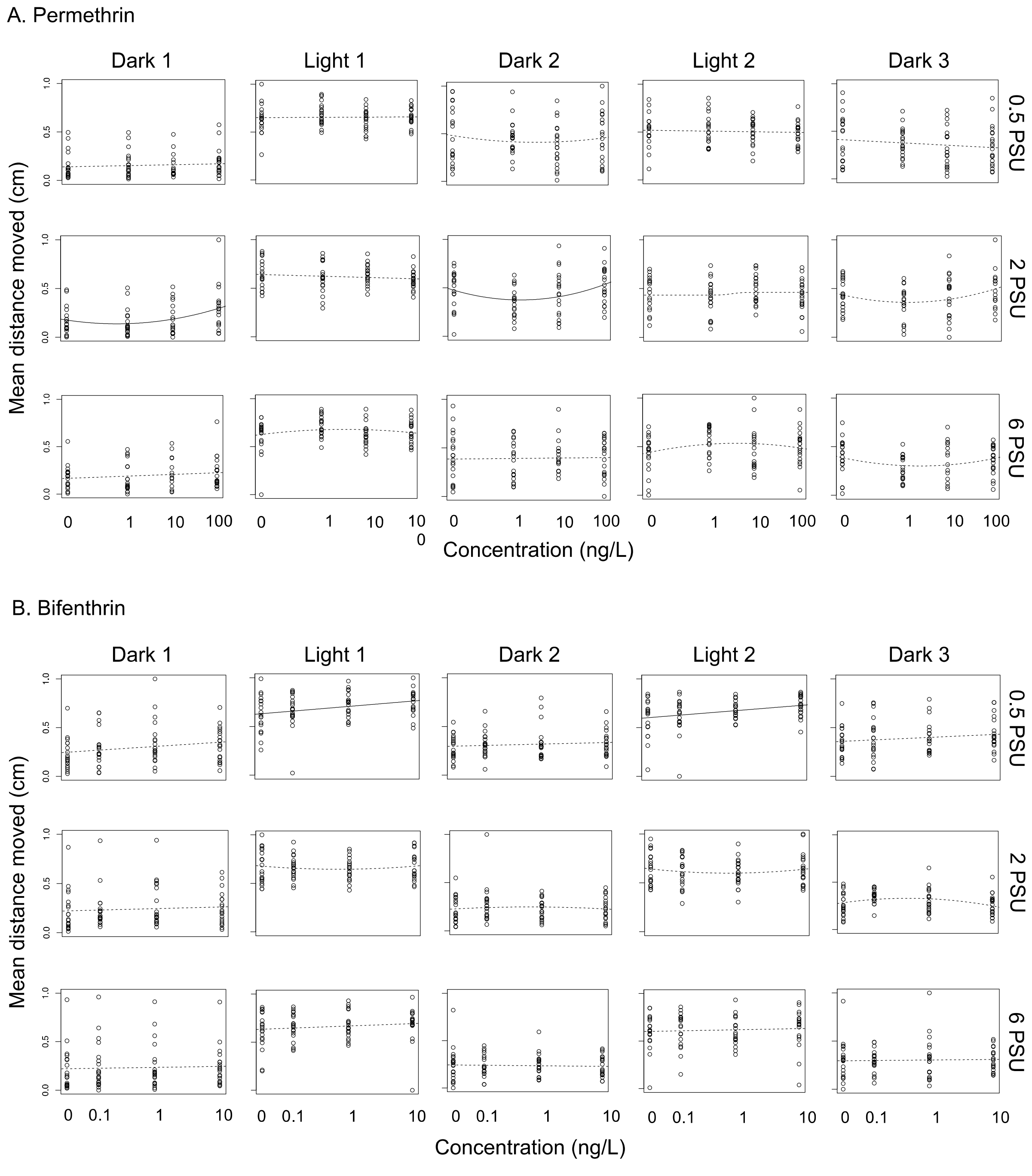

Figure S2. Dose responses within dark/light cycle across a salinity gradient (0.5, 2 and 6 PSU): (**A**) Delta Smelt larvae exposed to 0 (controls), 1, 10 and 100 ng/L permethrin, (**B**) Delta Smelt larvae exposed to 0 (controls), 0.1, 1 and 10 ng/L bifenthrin. Each dot represents the mean total distance moved of one individual, $n = 20$ larvae/treatment. Data was rescaled between 0 and 1 to facilitate comparison between periods, as well as pesticides. Five dose-response curves were fit using a maximum likelihood approach: linear, quadratic, sigmoidal, unimodal1, and unimodal2. Curves shown as a solid line are significantly better fits than a null intercept-only model ($p < 0.05$), curves shown as a dashed line are the best-fit of the five-curve option (lowest p-value), but not significantly better than the null model.
